# Factors affecting survival in White and Asian children with acute lymphoblastic leukaemia

**DOI:** 10.1054/bjoc.1999.1080

**Published:** 2000-04-03

**Authors:** J E Powell, E Mendez, S E Parkes, J R Mann

**Affiliations:** Department of Public Health & Epidemiology, University of Birmingham, Edgbaston, Birmingham, B15 2TT, UK; Department of Oncology, The Birmingham Children's Hospital NHS Trust, Steelhouse Lane, Birmingham, B4 6NH, UK

**Keywords:** childhood leukaemia, ALL, ethnic group, survival, deprivation, nutritional status

## Abstract

Some studies suggest that Asian children with leukaemia have a worse outcome than Whites. Survival of Asians with ALL treated at the Birmingham Children's Hospital from 1975 to 1994 was the same as that of Whites, despite their greater deprivation and poorer nutrition. For one 5-year period (1980–1984) Asians had significantly poorer survival, even after adjustment for prognostic factors. Poor treatment compliance during that period may have contributed to this difference. © 2000 Cancer Research Campaign


*British Journal of Cancer* (2000) **82**(9), 1568–1570
© 2000 Cancer Research Campaign
DOI: 10.1054/ bjoc.1999.1080, available online at http://www.idealibrary.com on IDEAL®

Short Communication

### Factors affecting survival in White and Asian children with acute lymphoblastic leukaemia

**JE Powell[1], E Mendez[2], SE Parkes[2] and JR Mann[2]**

[1]Department of Public Health & Epidemiology, University of Birmingham, Edgbaston, Birmingham B15 2TT, UK; [2] Department of Oncology, The Birmingham Children's Hospital NHS Trust, Steelhouse Lane, Birmingham B4 6NH, UK

**Summary** Some studies suggest that Asian children with leukaemia have a worse outcome than Whites. Survival of Asians with ALL treated at the Birmingham Children's Hospital from 1975 to 1994 was the same as that of Whites, despite their greater deprivation and poorer nutrition. For one 5-year period (1980–1984) Asians had significantly poorer survival, even after adjustment for prognostic factors. Poor treatment compliance during that period may have contributed to this difference. © 2000 Cancer Research Campaign

**Keywords:** childhood leukaemia; ALL; ethnic group; survival; deprivation; nutritional status

Acute lymphoblastic leukaemia (ALL) is the commonest childhood cancer in the develope world, and while around 70% of children survive (Burnet and Eden 1997), it is still a major cause of childhood mortality. Consequently, the identification of factors which influence survival (e.g. age, white cell count, immunophenotype), allowing tailoring of treatment, is an important approach to improving survival.

Ethnic origin is one factor which may affect survival. Studies from the USA (Walters et al, 1972; Pui et al, 1995) showed that in the 1970s and early 1980s Black children with ALL had poorer survival than Whites. This might have been due to the high proportion of poor prognosis disease found in Black children (Kalwinsky et al, 1985), but other factors such as social deprivation may have played a part. In developing countries, nutritional status has been shown to influence survival (Lobato-Mendizibal et al, 1989; Borato-Viana et al, 1994).

In the UK, Asians (i.e. originating from the Indian subcontinent) are the largest single ethnic minority group, comprising around 5% of the childhood population (OPCS, 1993). This group has been shown to be socially deprived and undernourished compared to their White counterparts (Rona and Chinn, 1986). Oakhill and Mann (1983) showed that Asian children treated in UKALL trials from 1972 to 1979 had a significantly poorer prognosis than their White counterparts, mainly due to deaths during remission. Since the Asians also had lower standardized height and weight scores, the authors concluded that lower socio-economic status, poor nutrition and difficulties in communication may have been responsible for the worse outcome. More recently, McKinney et al (1999) found that in Yorkshire, 41 Asian children with leukaemia diagnosed between 1974 and 1995 had poorer survival, even after adjustment for socio-economic status.

The Birmingham Children's Hospital (BCH) is the paediatric oncology referral centre for the West Midlands Region which contains almost 1 million children of whom 10% are Asians. We investigated the effects of prognostic factors, nutritional status and deprivation on the survival of White and Asian children treated at BCH between 1975 and 1994.

*Received 11 May 1999*
*Revised 29 September 1999*
*Accepted 1 October 1999*

*Correspondence to:* JE Powell

### METHODS

White and Asian children residing in the West Midlands Region who were diagnosed with ALL at the BCH between 1975 and 1994 were eligible for the study. Cases were identified from the records of the West Midlands Regional Children's Tumour Registry (WMRCTR). Information on treatment protocol, age, sex, white cell count, immunophenotype, height and weight at diagnosis was abstracted from hospital notes. Vital status was determined from the records of the WMRCTR, which actively follows up all registered cases.

The 20-year period was divided into four 5-year eras, within which different treatment protocols were employed in the hospital. Between 1975 and 1979 most children received the Medical Research Council's UKALL V protocol. Between 1980 and 1984, UKALL VIII was employed: this protocol, with its more intensive treatment, led to a marked improvement in outcome. Between 1985 and 1989, UKALL X was used, and UKALL XI from 1990 onwards.

Height and weight at diagnosis were used to derive the body mass index (BMI), a widely accepted index of nutritional status, which was standardized using contemporary age- and sex-specific reference data (Cole et al, 1995). Using the postcode at diagnosis, Townsend deprivation scores (Townsend et al, 1988) for the 1991 census enumeration district of residence were derived for each child. The Townsend score is derived from rates of unemployment, car and house ownership and overcrowding.

Chi-squared test and Mann–Whitney *U*-test were used to test for differences in patient characteristics. Linear regression was used to test for secular trends in deprivation scores and body mass index (BMI). Survival was analysed using Kaplan–Meier survival curves and Cox's proportional hazards regression.

**Table 1** Survival of 503 children with acute lymphoblastic leukaemia.

| Ethnic group | % surviving at 5 years (no. cases) White | Asian | Relative risk[a] of death (hazard ratio) Unadjusted RR[b] (95% CI) | P | Adjusted RR[c] (95% CI) | P |
|---|---|---|---|---|---|---|
| 1975–1979 | 44.1 (68) | 50.0 (8) | 0.82 (0.33–2.07) | 0.676 | 0.72 (0.27–1.85) | 0.478 |
| 1980–1984 | 65.3 (121) | 40.0 (10) | 2.34 (1.06–5.17) | 0.035 | 2.58 (1.11–5.97) | 0.027 |
| 1985–1989 | 76.5 (115) | 76.5 (17) | 1.01 (0.40–2.59) | 0.977 | 1.14 (0.44–2.97) | 0.785 |
| 1990–1994 | 77.5 (145) | 84.2 (19) | 0.69 (0.21–2.26) | 0.541 | 1.00 (0.29–3.40) | 0.878 |
| 1975–1994 | 69.0 (449) | 68.5 (54) | 1.12 (0.70–1.79) | 0.628 | 1.24 (0.77–1.99) | 0.371 |
| **Body mass index** | **Less undernourished** | **Most undernourished[d]** | | | | |
| 1975–1979 | 41.7 (60) | 64.3 (14) | 0.66 (0.31–1.42) | 0.288 | 0.63 (0.28–1.39) | 0.252 |
| 1980–1984 | 68.9 (103) | 47.8 (23) | 2.04 (1.13–3.66) | 0.018 | 1.55 (0.85–2.85) | 0.155 |
| 1985–1989 | 78.6 (103) | 74.1 (27) | 1.25 (0.59–2.65) | 0.563 | 0.90 (0.39–2.10) | 0.811 |
| 1990–1994 | 83.3 (137) | 63.6 (22) | 3.23 (1.48–7.02) | 0.003 | 3.23 (1.44–7.24) | 0.004 |
| 1975–1994 | 72.4 (403) | 62.7 (86) | 1.47 (1.03–2.08) | 0.033 | 1.13 (0.78–1.64) | 0.512 |
| **Deprivation score** | **Less deprived** | **Most deprived[e]** | | | | |
| 1975–1979 | 47.5 (59) | 35.3 (17) | 1.40 (0.74–2.64) | 0.296 | 1.50 (0.78–2.90) | 0.228 |
| 1980–1984 | 66.7 (108) | 47.8 (23) | 1.64 (0.90–2.99) | 0.107 | 1.25 (0.66–2.38) | 0.488 |
| 1985–1989 | 76.2 (105) | 77.8 (27) | 1.02 (0.47–2.22) | 0.958 | 1.10 (0.50–2.40) | 0.818 |
| 1990–1994 | 78.8 (133) | 75.8 (31) | 1.07 (0.47–2.44) | 0.876 | 1.00 (0.42–2.36) | 1.000 |
| 1975–1994 | 70.4 (405) | 62.9 (98) | 1.32 (0.94–1.87) | 0.111 | 1.22 (0.86–1.74) | 0.264 |

[a]Asian White cf. : most less cf. undernourished: most less cf. deprived. [b]Adjusted for era in the 1975–1994 analysis. [c]Adjusted for age (0, 1–4, 5–9, 10–14), WCC (<100, 100+), sex, immunophenotype (non-B-non-T vs. other); also for era in 1975–1994 analysis. [d]Age-standardized BMI –1.28 or below (the most undernourished 10% of the population). [e]Townsend Score +3.5 or higher (the most deprived quintile).

### RESULTS

Between 1975 and 1994, 449 White and 54 Asian children were treated for ALL at the Birmingham Children's Hospital. There were no significant differences between Whites and Asians for sex (Whites: 61% males, cf. 57% in Asians), white cell count (median $12.8 \times 10^9\ \mu l^{-1}$ in Whites, 21.2 in Asians), age at diagnosis (median age 4.5 years in Whites, 4.2 in Asians) or immunophenotype (B-cell, T-cell and unusual or poorly differentiated leukaemias occurring in 17% of Asians and Whites).

During the study period, 83.7% of Whites and 81.5% of Asians were entered into clinical trials. The proportion of children participating in trials did not increase over time (84% in 1975–1979; 89% in 1980–1984; 89% in 1985–1989; 82% in 1990–1994) and the proportion of White and Asian children in trials was similar for each of the four eras.

The median duration of follow-up (live cases) was 9.9 years (range 2–22 years). Five-year survival rates showed continuous improvement over time (44.7% in 1975–1979, 63.2% in 1980–1984, 76.5% in 1985–1989, 76.8% in 1990–1994). Table 1 shows survival rates according to ethnic group, nutritional status and deprivation score for each of the four eras. While there was no difference in survival between Asian and White children over the whole 20-year period, between 1980 and 1984 survival was significantly poorer in Asians. This difference remained after adjustment for other prognostic factors.

Table 1 also shows that undernourished children had significantly poorer survival throughout the 20-year study period, especially in the eras 1980–1984 and 1990–1994. However, after adjustment for other prognostic factors, only the effect for the period 1990–1994 remained significant.

No association was found between deprivation and survival, in either the unadjusted or adjusted analyses.

The lower survival of West Midlands Asian children in the era 1980–1984 is unlikely to have been caused by undernutrition or deprivation. While the overall BMI-score was slightly lower in Asians (–0.53 cf. –0.12 in Whites, $P = 0.09$), in the era 1980–1984 it was higher (+0.14 cf. –0.33 in Whites, $P = 0.30$). A significant improvement in BMI over the 20-year period was observed in Whites ($P = 0.024$) but not in Asians. Mean deprivation scores were higher in Asians in all four eras ($P < 0.005$), and scores did not alter significantly over time in either ethnic group.

We could not confirm Oakhill and Mann's (1983) finding that deaths in remission were common in the Asians. Of the seven Asian deaths from the 1980–1984 period, four relapsed on treatment, two failed to enter remission and one died of an accident while in first remission. During the 20-year period causes of death were similar in both ethnic groups (in both groups, 15% of deaths were treatment related while 7% of White and 5% of Asian deaths were due to other causes, including second malignancy).

Five of the ten Asians diagnosed from 1980 to 1984 had poor prognosis immunophenotypes (three T-cell, one B-cell, one with both lymphoid and myeloid markers) compared to 20% of the Whites ($P = 0.042$). This factor may account for much of the survival difference observed between Asians and Whites for that era. However, after adjusting for prognostic factors (immunophenotype, white cell count, age and sex) ethnic group remained an independent prognostic factor.

### DISCUSSION

A recent study from Yorkshire (McKinney et al, 1999) reported poorer survival among Asians diagnosed with leukaemia from 1974 to 1995. Our study over a similar time period does not confirm this. However, during the early 1980s, Asian children treated at BCH had significantly poorer survival than their White counterparts. Poor prognosis immunophenotypes were more common in Asians in that era, but the effect of ethnic group remained after adjusting for other prognostic factors. More recently, Whites and Asians have shown similar survival rates.

Pui et al (1995) demonstrated poorer survival in US Blacks between 1962 and 1983, but found no difference from 1984 to 1992. Since poor prognosis disease was more common in Blacks, they concluded that survival improvements were due to more effective therapies for high-risk disease. Although Pinkel (1995) suggested a role for improved nutrition, there was in fact no change in the proportion of undernourished Black children over time.

In our study, the improvement in Asian survival rate was not associated with improvements in nutritional status. While BMI significantly affected survival, this effect disappeared after adjustment for other prognostic factors, except in the most recent era, 1990–1994. Body mass index independently influenced survival in 128 Brazilian children (Borato-Viana et al, 1994), but Weir et al (1998) were unable to find any effect of BMI or ethnicity in their analysis of 1025 UK children treated on the MRC-UKALL X protocol. Our study also found no effect of ethnicity or BMI during the era 1985–1989 when UKALL-X was in use. One could speculate that any link between BMI or ethnicity and outcome is protocol-specific, though our findings could also be caused by the small numbers in our study.

McKinney et al (1999) observed a trend between increasing levels of social deprivation and poor leukaemia survival, though this effect disappeared after adjusting for other prognostic factors. While the Asians in our study had higher deprivation scores than Whites in all the four eras, we found no link between deprivation and survival.

In our study, survival was analysed over a 20-year period, and also in 5-year subsets (eras). It has been argued (Altman, 1991) that with multiple testing, adjustments should be made to the *P*-values. After applying the Bonferroni correction (each *P*-value is multiplied by 4– the number of tests) to the *P*-values for the 5-year eras in Table 1, only the effect of BMI in the 1990–1994 era remains significant ($P$ = 0.016). While these techniques are very conservative (Altman, 1991), and therefore of limited usefulness, they suggest that the ethnic survival difference observed in our data could be due to chance.

If it is not a chance finding, the question remains as to why Asian survival was so much poorer (40% alive at 5 years cf. 65% in Whites) during the era 1980–1984, and why it subsequently improved. Differences in treatment protocols are unlikely since an equal proportion of Whites and Asians were entered into current clinical trials. Much of the difference may be due to the large proportion of poor-prognosis phenotypes in the Asians from this era, but an ethnic effect remained after adjustment for this and other prognostic factors. Since the continuation of maintenance therapy for 2 years after diagnosis is known to improve survival (Rothwell, 1982), poor compliance might be the reason. Following the publication of Oakhill and Mann's paper in 1983 showing poorer prognosis in UK Asian children, efforts were made at the hospital to improve communications with ethnic minority parents. As well as providing explanatory leaflets in Asian languages, spoken information was also recorded onto cassette tapes for the benefit of illiterate parents. These measures, designed to improve treatment compliance, may have contributed to the observed improvements in survival.

In the past, UK ethnic minority groups have undoubtedly suffered from health inequalities, possibly due to social deprivation. Nowadays, with better integration into the community and more awareness in the health services of ethnic minority special needs, some of these problems may have been overcome. Even though West Midlands Asian children with ALL remain more deprived and marginally less well nourished than their White counterparts, there is evidence that their prognosis is now just as good as that of White children.

### ACKNOWLEDGEMENTS

We wish to thank Dr N Shaw, Consultant Paediatric Endocrinologist at BCH for help with the standardization of BMIs. The WMRCTR is funded by the Special Trustees for the Former United Birmingham Hospital Trust Funds. Dr Mendez was funded by a grant from the International Resource Development and the British Council.

### REFERENCES

Altman DG (1991) *Practical Statistics for Medical Research*. Chapman and Hall: London.

Borato Viana M, Murao M, Ramos G, Oliviera HM, de Carvalho RI, de Bastos M, Colosimo EA and Silvestrini WG (1994) Malnutrition as a prognostic factor in lymphoblastic leukaemia: a multivariate analysis. *Arch Dis Child* **71**: 304–310

Burnet AK and Eden OB (1997) The treatment of acute leukaemia. *Lancet* **349**: 270–275

Cole TJ, Freeman JV and Preece MA (1995) Body mass index reference curves for the UK, 1990. *Arch Dis Child* **73**: 25–29

Kalwinsky DK, Rivera G, Dahl GV, Roberson P, George S, Murphy SB and Simone JV (1985) Variation by race in presenting clinical and biological features of childhood acute lymphoblastic leukaemia: implications for treatment outcome. *Leuk Res* **9**: 817–823

Lobato-Mendizabal E, Ruiz-Arguelles GJ and Marin-Lopez A (1989) Leukaemia and nutrition I: malnutrition is an adverse prognostic factor in the outcome of treatment of patients with standard-risk acute lymphoblastic leukaemia. *Leuk Res* **13**: 899–906

McKinney PA, Feltbower RG, Parslow RC, Lewis IJ, Picton S, Kinsey SE and Bailey CC (1999) Survival from childhood cancer in Yorkshire, UK: effect of ethnicity and socio-economic status. *Eur J Cancer* **35**: 1816–1823

Oakhill A and Mann JR (1983) Poor prognosis of acute lymphoblastic leukaemia in Asian children living in the United Kingdom. *Br Med J* **286**: 839–841

OPCS (Office of Population Censuses and Surveys) (1993) *1991 Census Report for England: Regional Health Authorities*. HMSO: London

Pinkel D (1995) Acute lymphoid leukaemia outcomes in black and white children (letter). *JAMA* **274**: 379–380

Pui CH, Boyett JM, Hancock ML, Pratt CB, Meyer WH and Crist WM (1995) Outcome of treatment for childhood cancer in black as compared with white children. *JAMA* **273**: 633–637

Rona RJ and Chinn S (1986) National Study on Health and Growth: social and biological factors associated with height of children from ethnic groups living in England. *Ann Hum Biol* **13**: 453–471

Rothwell D (1982) Duration of chemotherapy in childhood acute lymphoblastic leukaemia. *Med Pediatr Oncol* **10**: 511–520

Townsend P, Phillimore P and Beattie A (1988) *Health and Deprivation. Inequality and the North*. Croom Helm: London

Walters TR, Bushmore M and Simone J (1972) Poor prognosis in negro children with acute lymphoblastic leukaemia. *Cancer* **29**: 210–214

Weir J, Reilly JJ, McColl JH and Gibson BES (1998) No evidence for an effect of nutritional status at diagnosis on prognosis in children with acute lymphoblastic leukaemia. *J Paediatr Hematol Oncol* **20**: 534–538

 *© 2000 Cancer Research Campaign*

